# Red Light Triggers Lesion Formation in the *sdr7-6* Mutant of Rice

**DOI:** 10.3390/plants15030490

**Published:** 2026-02-05

**Authors:** Yanmei Zheng, Yanjia Xiao, Feihe Chen, Xi Luo, Minrong Jiang, Linyan Wei, Jinglan Wang, Haomin Zhang, Yidong Wei, Lili Cui, Yongsheng Zhu, Hongguang Xie, Qiuhua Cai, Huaan Xie, Jianfu Zhang

**Affiliations:** 1 Rice Research Institute, Fujian Academy of Agricultural Sciences, Fuzhou 350003, China; zhengyanmei@faas.cn (Y.Z.); joe310121@163.com (Y.X.); chenfeihe0301@163.com (F.C.); luoxi@faas.cn (X.L.); jmr987654321@163.com (M.J.); weilinyan@faas.cn (L.W.); wangjinlan07@163.com (J.W.); zhmin1325@163.com (H.Z.); weiyidong@faas.cn (Y.W.); guzai0429@126.com (L.C.); frwenj@126.com (Y.Z.); xiehongguang@faas.cn (H.X.); caiqiuhua@163.com (Q.C.); huaanxie@163.com (H.X.); 2 Key Laboratory of Germplasm Innovation and Molecular Breeding of Hybrid Rice for South China, Ministry of Agriculture and Affairs P.R. China/Incubator of National Key Laboratory of Germplasm Innovation and Molecular Breeding Between Fujian and Ministry of Sciences and Technology/Fuzhou Branch, National Rice Improvement Center of China/Fujian Engineering Laboratory of Crop Molecular Breeding/Fujian Key Laboratory of Rice Molecular Breeding, Fuzhou 350003, China

**Keywords:** rice, lesion formation, *sdr7-6*, short chain alcohol dehydrogenase/reductase, red light

## Abstract

Lesion mimic mutants are ideal materials for investigating programmed cell death in plants. The rice mutant *sdr7-6* exhibits a light-dependent lesion mimic phenotype, but the specific light conditions that trigger necrosis and the underlying molecular mechanisms remain unclear. In this study, we characterized the light-dependent necrotic phenotype of *sdr7-6* under red, blue, and far-red light. We found that lesion formation was exclusively triggered by red light and occurred independently of light intensity or photoperiod. Knockout of *phyB* markedly alleviated lesion development, confirming the role of phyB-mediated red-light signaling in this process. Physiological analyses revealed a significant reduction in photosynthetic capacity in *sdr7-6*, primarily due to disrupted chloroplast ultrastructure. Consistent with these findings, transcriptome profiling indicated strong downregulation of genes associated with chloroplast function, photosynthesis, and light responses. Together, these results demonstrate that SDR7-6 functions as a previously unrecognized downstream component of phyB-mediated red-light signaling and is involved in red-light-dependent lesion formation in rice, which is tightly associated with compromised chloroplast integrity and function. This work provides new insights into the molecular mechanisms underlying red-light perception and its regulation of lesion formation in rice.

## 1. Introduction

Lesion mimic mutants exhibit spontaneous cell death resembling pathogen-inducible, hypersensitive cell death. These mutants provide excellent materials for studying programmed cell death (PCD) and how it is coordinated with plant growth [1,2]. The initiation of necrotic spots is extremely complex and is regulated by disease resistance pathways, cell death regulators, metabolic enzymes and external environmental factors [2,3]. External environmental factors include light, temperature and humidity [3,4].

Light, as an important abiotic stress factor, regulates the initiation of lesion formation through photoperiod, light intensity, and light quality. The *glp1* mutant exhibited UV-B-dependent lesions [5]. The *cat2* mutant develops lesions in a manner that depends on day length and light intensity [6]. Excessive white light or red light can cause cell death in *lsd1* mutant [7]. Chloroplasts, the primary sites of photosynthesis, are essential for light energy capture and carbon assimilation. In many lesion mimic mutants, chlorophyll content is reduced, chloroplast ultrastructure is disrupted, photosynthetic capacity consequently declines, and reactive oxygen species (ROS) accumulate. Transcriptome analysis has revealed extensive reprogramming of genes associated with stress adaptation, secondary metabolism, antioxidant pathways, and photosynthesis [8,9].

Light-dependent cell death is a plant-specific type of programmed cell death, and its requirement for light is commonly associated with the generation of reactive oxygen species (ROS) during photosynthesis. This ROS burst leads to chloroplast destruction and subsequently initiates the hypersensitive response (HR) and PCD, ultimately resulting in lesion formation. However, the specific signaling pathways and photoreceptors involved differ significantly across light spectra. In the *flu* mutant, singlet oxygen is necessary but insufficient to trigger cell death; full execution of the PCD response requires blue-light-dependent activation of the photoreceptor CRY1, which enables the transcriptional reprogramming required for lesion formation [10]. Ultraviolet (UV) radiation is composed of UV-A (320–400 nm), UV-B (280–320 nm), and UV-C (200–280 nm) [11], and the current understanding of plant UV responses comes largely from studies on UV-B. At low fluence rates, UV-B signaling is partially mediated through the UV RESISTANCE LOCUS 8 (UVR8) photoreceptor in *Arabidopsis*. At higher levels, various regulatory mechanisms are involved, including UVR8-independent pathways, ROS-driven oxidative stress, and cellular damage [12,13,14,15]. In the rice *glp1* mutant, loss of OsGLP1 suppresses the UVR8 pathway and overactivates the MAPK pathway, leading to impaired photosynthesis and excessive programmed cell death, which results in lesion mimic formation under UV-B stress [5]. Overexposure to UV-C induces PCD through caspase-like protease activation, oligonucleosomal DNA fragmentation, and apoptotic nuclear morphology [11]. In the mechanism of red-light-triggered necrosis, treatment with 3-(3,4-dichlorophenyl)-1,1-dimethylurea (DCMU) reduced cell death in the *lsd1* mutant, indicating that the redox status of the plastoquinone (PQ) pool is involved in red-light-induced PCD in *lsd1*. By preventing electron transfer from PSII to the PQ pool, DCMU likely suppresses PQ over-reduction and subsequent reactive oxygen species (ROS) production, thereby attenuating cell death. Under red-light conditions, the *lsd1* mutant exhibited enhanced cell death, whereas the *hy5-215* mutant suppressed cell death, suggesting that HY5-dependent red-light signaling is required for the full activation of this PCD pathway [7]. Taken together, the mechanisms underlying light-induced cell death differ among light types, motivating further investigation into the specific light conditions that regulate lesion formation.

In a previous study, we identified a new rice lesion mimic mutant, *sdr7-6*, which encodes a putative short-chain alcohol dehydrogenase/reductase. Its lesion mimic phenotype is light-dependent [16]. Based on this observation, we hypothesized that light acts as a trigger for lesion development in *sdr7-6*, initiating downstream events leading to programmed cell death. In the present study, we used this mutant to investigate the specific light conditions that trigger lesion formation and to elucidate the transcriptional basis of necrosis. Our results improve our understanding of the crosstalk between light signaling and immune- or PCD-related pathways and provide a foundation for future studies on phyB-mediated signaling. These insights may also help balance disease resistance and growth, offering potential avenues for future crop improvement.

## 2. Results

### 2.1. The sdr7-6 Mutant Showed Enhanced Sensitivity to Red Light

**Figure 1:**
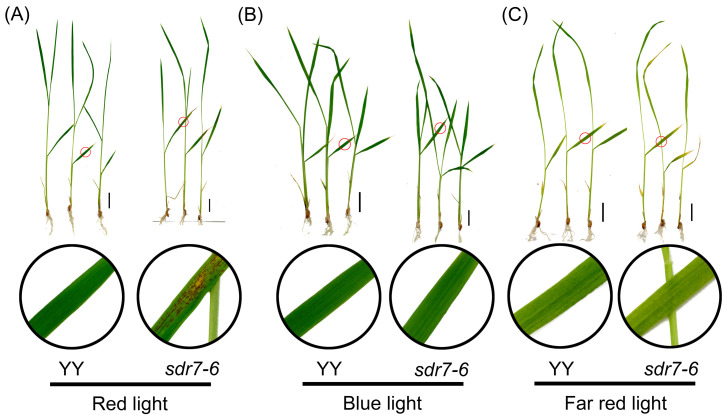
Impact of full-intensity red-, blue-, and far-red-light treatments on lesion formation. (**A**) Phenotype of wild-type YY and *sdr7-6* mutant under full-intensity red-light treatment (277.8 μmol m^−2^ s^−1^). (**B**) Phenotype of wild-type YY and *sdr7-6* mutant under full-intensity blue-light treatment (245.2 μmol m^−2^ s^−1^). (**C**) Phenotype of wild-type YY and *sdr7-6* mutant under full-intensity far-red-light treatment (363.5 μmol m^−2^ s^−1^). The region highlighted by the red circle in the upper panel is shown at higher magnification below. Scale bar = 2 cm.

In many lesion mimic mutants, lesion onset is modulated by light quality, intensity or photoperiod. We previously demonstrated that necrotic-spot formation in *sdr7-6* is light-dependent, and no lesions developed when plants were kept under shading conditions [16]. Which specific wavelengths trigger the lesions, and how they do so, remained unresolved. Brown spots appeared at the four-leaf stage when plants were grown in a controlled chamber (28 °C, 70% relative humidity) under a 16 h light/8 h dark photoperiod, equipped with light-emitting diodes (JIUPO-1WSLED-210). Spectral analysis showed that red and blue wavelengths dominated the output (Supplementary Figure S1), with a total photon flux density (PFD) of 201.9 μmol m^−2^ s^−1^, of which the individual PFDs were 118.5, 45.0, and 4.9 μmol m^−2^ s^−1^ for red, blue, and far-red light, respectively. To test the effects of individual wavebands, three-leaf-stage seedlings were transferred to chambers (JIUPO-BPC500H) equipped with light-emitting diodes (JPBIO-1WFLED-216) providing either red, blue or far-red light under a 16 h light/8 h dark photoperiod at 70% relative humidity. The total PFD under these monochromatic light treatments was higher than that under the JIUPO-1WSLED-210 condition. Necrotic spots appeared after 2 days of full intensity (Figure 1A), whereas no spots appeared under blue light (Figure 1B). Since far-red-light treatment can counteract red-light responses [17], we asked whether it would suppress spot formation. In line with our hypothesis, seedlings treated with far-red light showed no lesion formation (Figure 1C). These results indicate that red light alone is sufficient to trigger lesion formation in *sdr7-6*.

### 2.2. Red Light-Induced Lesion Formation Is Independent of Light Intensity and Photoperiod

**Figure 2:**
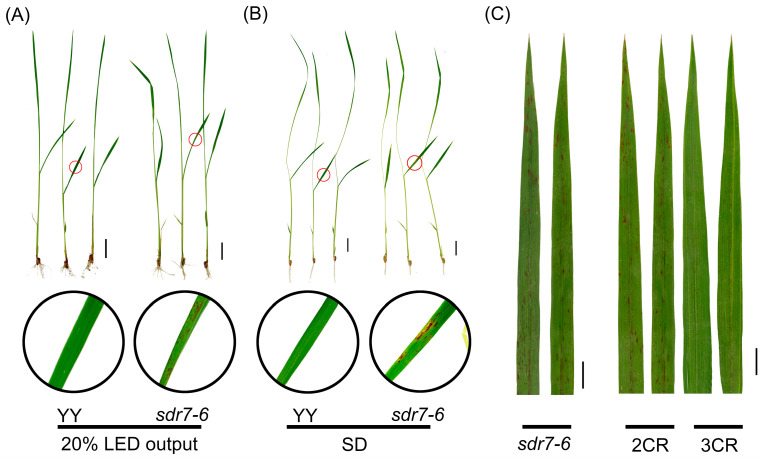
Impact of light intensity and short-day conditions on lesion formation. (**A**) Phenotype of wild-type YY and *sdr7-6* mutant under 20% LED-output red-light (65.5 μmol m^−2^ s^−1^). (**B**) Phenotypes of wild-type YY and *sdr7-6* mutant under short-day (SD) conditions (6 h red light/18 h dark). The region highlighted by the red circle in the upper panel is shown at higher magnification below. (**C**) Phenotypes of *phyA*, *phyB*, and *phyC* knockout mutants. 2 CR indicates the *phyA*/*phyC* double knockout in *sdr7-6* background, and 3 CR indicates the *phyA*/*phyB*/*phyC* triple knockout in *sdr7-6* background. Each leaf represents a different line. Scale bar = 2 cm.

To determine whether reduced light intensity affects lesion formation, we exposed three-leaf-stage seedlings to 20% of full red-light intensity under the same growth conditions as the full-intensity treatment. Although lesion formation was delayed compared to the full-intensity treatment, this reduced light intensity was still sufficient to induce lesions (Figure 2A). To assess the effect of photoperiod, seedlings at the same stage were subjected to a 6 h red-light/18 h dark cycle. The onset of lesions was observed on day 4. Lesions still developed under short-day treatment, indicating that photoperiod is not a determining factor for lesion formation in the *sdr7-6* mutant (Figure 2B).

### 2.3. The sdr7-6 Mutation Did Not Affect the Subcellular Localization of phyB

Red- and far-red-light signals are perceived by the phytochrome (phy) family of photoreceptors. In rice, three phytochromes—phyA, phyB, and phyC—have been identified [18]. Among them, phyB serves as a primary receptor [19]. To further determine whether red light triggers lesion formation, we knocked out the photoreceptors *phyA*, *phyB*, and *phyC*. As shown in Figure 2C, the *phyA/phyC* double knockout exhibited a lesion phenotype similar to that of *sdr7-6*, whereas the *phyA/phyB/phyC* triple knockout markedly alleviated the lesion phenotype and showed reduced sensitivity to red light. These results provide further evidence that phyB functions as the primary red-light receptor regulating lesion formation in the *sdr7-6* mutant.

**Figure 3:**
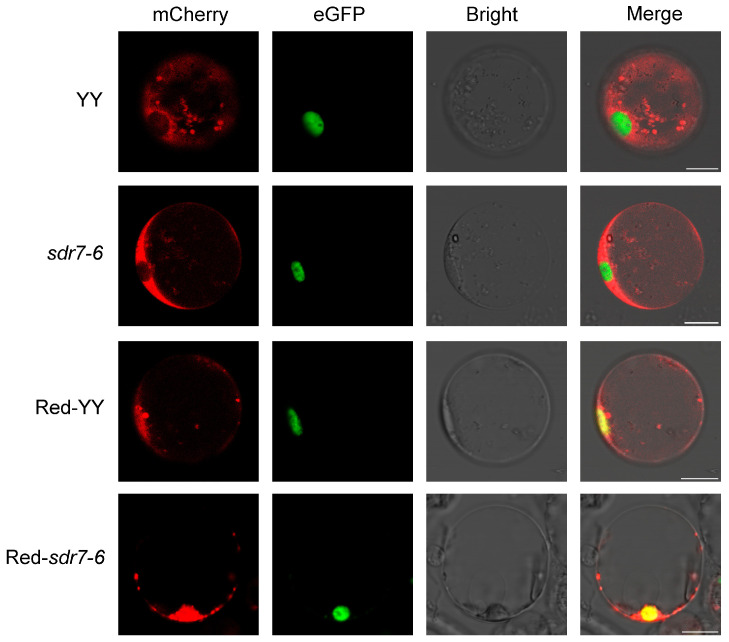
Subcellular localization of phyB in protoplasts of wild-type (YY) and *sdr7-6* under dark and red-light treatments. phyB-mCherry (red) was co-transformed with the nuclear-localized marker OsbZIP72-eGFP (green). Merged images represent the overlay of mCherry, eGFP, and bright-field signals. YY and *sdr7-6* denote protoplasts incubated in darkness for 15 h, whereas Red-YY and Red-*sdr7-6* denote protoplasts after 15 h of red-light treatment. Scale bar = 10 μm.

Under red-light exposure, phyB converts from Pr (red-absorbing form) to Pfr (far-red-absorbing form) and translocates from the cytosol to the nucleus to initiate downstream signaling [20]. To determine whether the *sdr7-6* mutation affects the subcellular localization of phyB, plasmids encoding p35S::phyB-mCherry and p35S::OsbZIP72-eGFP were cotransformed into protoplasts derived from wild-type YY and the *sdr7-6* mutant, respectively. The p35S::OsbZIP72-eGFP was used as a nuclear localization marker to assess the nuclear localization of phyB. Protoplasts were incubated in darkness or exposed to red light for 15 h. When *sdr7-6* protoplasts were maintained in darkness, phyB localized predominantly in the cytosol, whereas under red-light conditions, phyB was observed in the nucleus and colocalized with OsbZIP72 (Figure 3). These observations indicate that the *sdr7-6* mutation does not impair the red-light-induced relocalization of phyB.

### 2.4. Photosynthetic Capacity Was Reduced in sdr7-6

**Figure 4:**
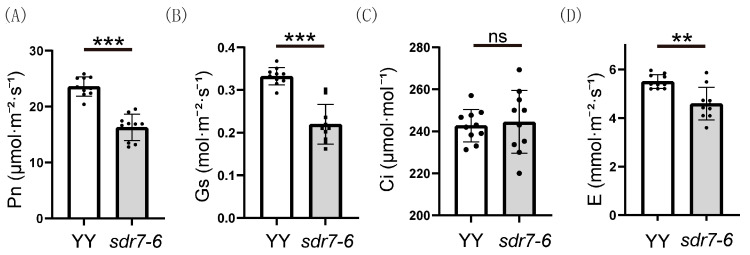
Photosynthetic capacity of wild type and *sdr7-6* mutant. (**A**) Net photosynthetic rate (Pn); (**B**) Stomatal conductance (Gs); (**C**) Intercellular CO_2_ concentration (Ci); (**D**) Transpiration rate (E). Measurements were performed on 10 independent leaves per genotype. The experiment was conducted twice. Data from one representative experiment are shown. Values are presented as means ± SD. Statistical significance was determined by Student’s *t*-test (** *p* < 0.01; *** *p* < 0.001; ns, not significant).

We previously reported that chlorophyll content was reduced in lesion-bearing leaves of *sdr7-6* [16], indicating that photosynthesis might be affected. To verify this hypothesis, we further analyzed the photosynthetic capacity of wild-type and *sdr7-6* leaves. As shown in Figure 4, the net photosynthetic rate (Pn), stomatal conductance (Gs) and transpiration rate (E) were significantly reduced in the mutant. The intercellular CO_2_ concentration (Ci) showed no significant difference between WT and the mutant (Figure 4C).

### 2.5. sdr7-6 Chloroplasts Exhibited Abnormal Morphology

**Figure 5:**
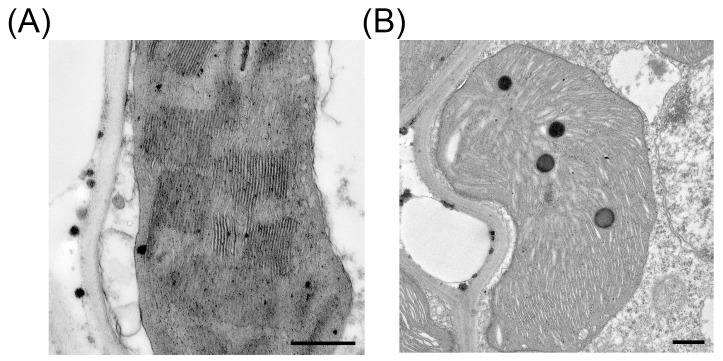
*sdr7-6* chloroplasts exhibit disrupted thylakoid structures. (**A**) Ultrastructure of chloroplast in wild-type YY. (**B**) Ultrastructure of chloroplast in *sdr7-6* mutant. Scale bar = 500 nm.

In many lesion mimic mutants, the chloroplast structure is irregular [9,21,22]. The organization of grana in both lateral and vertical dimensions is essential for the proper operation, regulatory control, and maintenance of the photosynthetic apparatus responsible for energy conversion [23]. Since Ci showed no significant difference between the wild type and the mutant, the reduction in photosynthetic rate was unlikely due to CO_2_ limitation caused by stomatal closure, but rather to the decline in the photosynthetic activity of mesophyll cells. We therefore examined the ultrastructural changes in the *sdr7-6* mutant using transmission electron microscopy (TEM). In wild-type YY, grana stacks were well maintained (Figure 5A), whereas in *sdr7-6* the thylakoid membranes became indistinct and the grana stacks were no longer visible (Figure 5B). These changes are consistent with the reduced photosynthetic capacity.

### 2.6. The Expression of Light-Responsive Genes Was Significantly Altered in sdr7-6

To explore the transcriptional basis underlying lesion formation, we performed global transcriptome profiling of the *sdr7-6* mutant exhibiting spontaneous lesions and the wild-type YY. Using thresholds of log_2_FC ≥ 1, q-value < 0.05, and FPKM > 0.5 in all three replicates under at least one condition, we identified 1030 differentially expressed genes (DEGs) between the *sdr7-6* mutant and the wild type.

**Figure 6:**
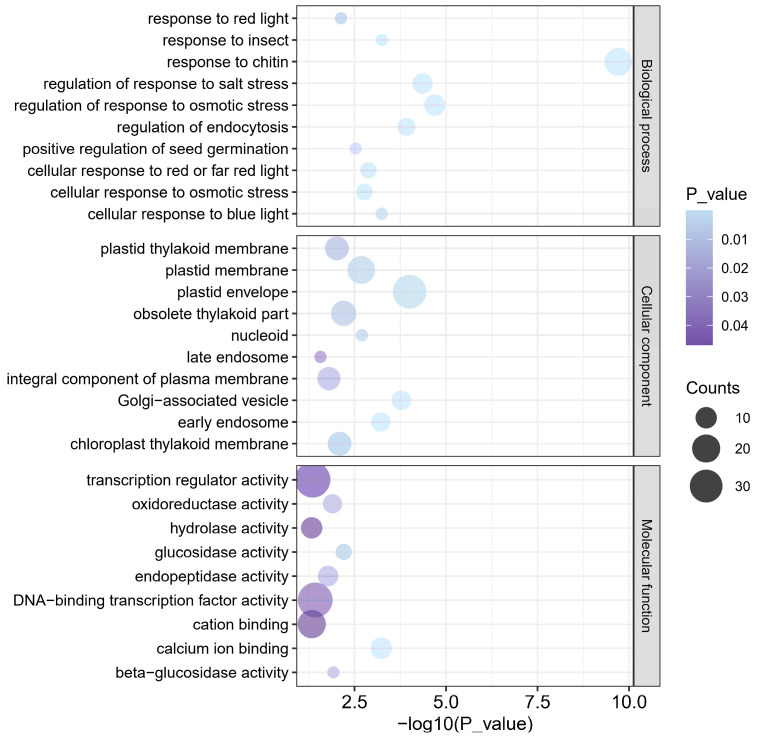
GO term enrichment analysis of DEGs between wild type and *sdr7-6*.

To gain further insights into the biological functions associated with the DEGs, we conducted Gene Ontology (GO) enrichment analysis. The top 10 enriched GO terms in both the cellular component and biological process categories were predominantly associated with the chloroplasts and light stimuli (Figure 6). To verify the reliability of the data, a subset of randomly selected genes was validated by qRT-PCR analysis. Genes associated with GO cellular component terms related to photosynthesis and chloroplasts were significantly downregulated (Supplementary Figure S2). The expression levels of four plastid ribosomal large-subunit proteins (PRPL3, PRPL4, PRPL5 and WLP1), cytochrome P450 (CYP97B4), a core component of the chloroplast RNA editing complex (MORF9), and the accumulation and replication of chloroplasts 5 (ARC5) and light-regulated gene 1 (LIR1) were significantly reduced in the *sdr7-6* mutant.

Specifically, GO enrichment analysis of the biological process category indicated that genes responsive to red, blue, and far-red light were significantly enriched. Among these enriched genes, a chlorophyll a-b binding protein gene (*Os09g0439500*) was strongly downregulated in *sdr7-6*, while three early light-induced protein genes (*Os01g0246400*, *Os07g0178700*, *Os07g0178800*) and the light-responsive regulator OsBBX2 were significantly upregulated (Supplementary Figure S3).

## 3. Discussion

Abiotic factors, particularly light and temperature, are widely recognized as critical determinants of lesion formation [24]. Various lesion mimic mutants exhibit diverse light-dependent phenotypes. For example, mutation in *OsGLP1* causes a UV-B-dependent lesion mimic phenotype [5], whereas the *flu* mutant requires blue light to trigger programmed cell death [10]. In the present study, we exposed *sdr7-6* plants to red, blue, and far-red light. Red light induced lesion formation, whereas no lesions developed under blue or far-red light. These results indicate that red light is sufficient to trigger lesions, consistent with observations in the *psi2* mutant [25]. The *sdr7-6* mutant does not affect the pathways mediating lesion formation under blue or far-red light.

Previous studies have shown that high light intensity can induce lesion formation in *lsd1* [26] and exacerbate lesion development in rice leaves [27]. In our experiments, however, lesions were triggered in *sdr7-6* even when red-light intensity was reduced to 20% of the full level, indicating that light intensity is not a limiting factor for lesion formation in this mutant. Nonetheless, higher light intensity accelerates lesion onset, whereas reduced light delays it, a phenomenon also reported in the *acd2* mutant [28]. Unlike the long-day-induced lesion formation reported in *lsd1* [26,29], lesion formation in *sdr7-6* occurred independently of photoperiod, developing under both short-day and long-day conditions, and the mutant also displayed enhanced sensitivity to red light.

The diversity of light-induced lesion formation highlights the complexity of its regulatory mechanisms. Alterations in chloroplast ultrastructure, the disappearance of grana stacking, decreased chlorophyll content, reduced photosynthetic capacity, and accumulated ROS are common characteristics of lesion mimic mutants, and similar changes were observed in our material. Knockout of *phyB* alleviated lesion formation in *sdr7-6*, indicating that this process is mediated by the phyB signaling pathway. Under red-light treatment, phyB nuclear translocation remained unchanged in the *sdr7-6* background, demonstrating that the lesion phenotype does not result from defective phyB nuclear localization. Instead, SDR7-6 likely acts as a downstream modulator of phyB signaling to regulate red-light-triggered cell death responses. Red-light-induced programmed cell death (PCD) in *Arabidopsis* is antagonistically regulated by LSD1 and HY5. In the *lsd1* mutant, the absence of LSD1 leads to elevated ROS and SA levels, resulting in enhanced PCD, whereas HY5 promotes PCD by directly interacting with the red-light receptor phyB. Consistently, the *hy5-215* mutant exhibits reduced cell death in the *lsd1* background. In our study, knockout of *phyB* in the *sdr7-6* mutant similarly reduced PCD, which aligns with the phenotype observed in *hy5-215*. Previous results showed that ROS and SA levels were elevated in *sdr7-6* [16], suggesting that the regulation of red-light-induced cell death in *sdr7-6* may be similar to that in *lsd1* in *Arabidopsis.* Collectively, these findings support a regulatory framework in which red-light-induced programmed cell death is controlled by a balance between positive and negative signaling components within the phyB-mediated red-light signaling network. SDR7-6 may serve as a negative regulator, playing a role comparable to that of LSD1 in restraining excessive ROS- and SA-associated cell death under red-light conditions. Loss of this regulatory constraint in *sdr7-6* results in heightened sensitivity to red light and uncontrolled lesion formation.

Investigation of gene expression patterns and their functional enrichment provides key insights into molecular responses. Although lesion mimic mutant genes differ widely in type and pathway, the downstream immune responses and molecular events leading to cell death are generally conserved [30]. Genes related to photosynthesis, chloroplast function, light signaling, and redox balance are commonly enriched in lesion mimic mutants [24]. In this study, significant GO enrichment of chloroplast- and photosynthesis-related genes, as well as genes responsive to red, blue, and far-red light, provides a molecular explanation for the observed phenotypes in *sdr7-6*, including impaired chloroplast ultrastructure, reduced photosynthetic capacity, and necrotic lesions in response to red light. Enrichment of oxidoreductase activity further indicates that disruption of chloroplast-related processes may lead to excessive ROS production, as previously demonstrated in lesion mimic mutants. Our study sheds light on the role of red-light perception in regulating the molecular pathways underlying lesion formation in rice. However, the direct factors interacting with SDR7-6 remain unclear, and future studies should aim to elucidate how SDR7-6 participates in the phyB-mediated red light signaling pathway.

## 4. Materials and Methods

### 4.1. Plant Material and Growth Conditions

The rice material *Yunyin* (YY), a local landrace from Yunnan Province, and the *sdr7-6* mutant, which was derived from YY through EMS mutagenesis (*Oryza sativa* L. ssp. *japonica*), were used in this study. All plants were grown either in natural paddy fields in Fuzhou (Fujian Province, China) or in a controlled-growth chamber (28 °C, 70% relative humidity) under a 16 h light/8-h dark photoperiod, equipped with light-emitting diodes (JIUPO-1WSLED-210, Fujian Jiupo Biotechnology, Fuzhou, China). Plants were positioned 55 cm below the panel, where the total photon flux density (PFD) was 201.9 μmol m^−2^ s^−1^, with blue-, red-, and far-red-light components of 45, 118.5, and 4.93 μmol m^−2^ s^−1^, respectively. Photon flux density was measured using a spectroradiometer (PG100N, UPRtek, Zhunan, Taiwan).

### 4.2. Illumination Treatments

Rice seedlings were cultivated in Yoshida medium (Coolaber) with 16/8 h light/dark cycle. Different illumination treatments were conducted at the three-leaf-stage of rice seedlings in climate-controlled plant growth chambers (JIUPO-BPC500H, Fujian Jiupo Biotechnology, Fuzhou, China), equipped with light-emitting diodes (JPBIO-1WFLED-216, Fujian Jiupo Biotechnology, Fuzhou, China). At a seedling-to-panel distance of 25.5 cm and 100% output (defined as full intensity in this study), the PFDs of blue, red, and far-red light were 245.2, 277.8, and 363.5 μmol m^−2^ s^−1^, respectively, under a 16 h light/8 h dark photoperiod. The chamber conditions were maintained at 28 °C with 70% relative humidity. For low-light intensity treatment, the output was reduced to 20%. At a seedling-to-panel distance of 25.5 cm, the PFD of red light was 65.5 μmol m^−2^ s^−1^. For short-day (SD) treatment, 100% output was used, and seedlings were cultivated under 6 h red light/18 h dark at 28 °C and 70% relative humidity.

### 4.3. Knockout Vector Construction

Double knockout lines of *phyA*/*phyC* were generated using CRISPR/Cas9 system [30]. Triple knockout lines of *phyA*/*phyB*/*phyC* were generated using CRISPR/Cas9 system [31]. The primers for the construction of CRISPR/Cas9 lines are listed in Supplementary Table S1.

### 4.4. Subceellular Localization of phyB

To determine the subcellular localization of phyB in wild-type YY and *sdr7-6* mutant protoplasts, the *phyB* coding sequence lacking the stop codon was amplified and inserted into the mCherry-tagged 583 vector (XbaI/SmaI) to generate a phyB-mCherry fusion construct. The constructed vector was co-transformed with the nuclear-localized marker OsbZIP72-eGFP into wild-type YY and *sdr7-6* mutant protoplasts, respectively [32]. Protoplasts were incubated at 28 °C under dark or red-light conditions for 15 h, and the fluorescence signals were subsequently observed using a confocal laser scanning microscope (DMi8; Leica, Wetzlar, Germany).

### 4.5. Photosynthetic Capacity Measurements

Photosynthetic capacity was measured on fully expanded leaves of YY and lesion-bearing *sdr7-6* using a portable gas exchange fluorescence system LI-6400XT (LI-COR, Lincoln, NE, USA). In total, 10 leaves of each line were measured.

### 4.6. Electron Microscopy Analysis

For electron microscopy, the fresh leaves of YY and *sdr7-6* with necrosis were immersed in ice-cold 2.5% glutaraldehyde solution (pH 7.0) and infiltrated for 30 min, then prefixed at 4 °C overnight. The samples were subsequently fixed with 2% osmium tetroxide. The processed ultrathin samples were observed with transmission electron microscopy (FEI Tecnai Spirit G2 BioTWIN, Hillsboro, OR, USA).

### 4.7. Bioinformatics Analysis of RNA-Seq Data

Samples were collected from wild-type and *sdr7-6* plants exhibiting lesions, with three biological replicates per genotype. Total RNA was extracted and processed by the Beijing Genomics Institute (BGI). Differentially expressed genes (DEGs) were identified using thresholds of q-value < 0.05, |log_2_(fold change)| ≥ 1, and FPKM > 0.5 in all three replicates under at least one condition. Gene Ontology (GO) enrichment analysis was conducted using Tbtools (TBtools-II, v2.337) [33], and the corresponding GO enrichment plots were generated in R (version 4.5.1).

### 4.8. RNA Extraction and qRT-PCR Analysis

For each biological replicate, the third leaf from the top of five wild-type and *sdr7-6* plants bearing lesions was pooled, respectively. Total RNA was extracted with TransZol Up Plus RNA Kit (TransGen Biotech, Beijing, China). First-strand cDNA was synthesized with the ReverTra Ace qPCR RT Master Mix with gDNA Remover (TOYOBO, Osaka, Japan).

qRT-PCR was performed using ChamQ Universal SYBR qPCR Master mix (Vazyme, Nanjing, China) on a QuantStudio 6 Flex Real-Time PCR system (Thermo Fisher Scientific, Waltham, MA, USA). Genes related to photosynthesis, chloroplast function and light responses enriched in GO terms were selected for validation, including four plastid ribosomal large-subunit protein genes (*PRPL3*, *PRPL4*, *PRPL5*, *WLP1*), *CYP97B4*, *MORF9*, *ARC5*, *LIR1*, a chlorophyll a-b binding protein gene (*Os09g0439500*), three early light-induced protein genes (*Os01g0246400*, *Os07g0178700*, *Os07g0178800*), and the light-responsive regulator OsBBX2 (*Os02g0176000*).

qRT-PCR was conducted under the following conditions: initial denaturation at 95 °C for 30 s, followed by 40 cycles of denaturation at 95 °C for 10 s and annealing/extension at 60 °C for 30 s. The rice *Actin* gene was used as an internal control, and relative gene expression levels were calculated using the 2^−ΔΔCt^ method. The primers are listed in Supplementary Table S1.

## 5. Conclusions

Lesion formation in *sdr7-6* was triggered exclusively by red light and occurred independently of light intensity or photoperiod. The lesions exhibited reduced photosynthetic capacity due to impaired mesophyll cell activity, consistent with abnormal chloroplast ultrastructure and transcriptome data showing significant downregulation of light- and photosynthesis-related genes. Based on our results, we propose a working model in which red light activates phyB signaling, leading to ROS accumulation, and SDR7-6 may function downstream of phyB to scavenge ROS and prevent inappropriate activation of HR, thereby controlling lesion formation in rice. Further studies are required to identify the direct molecular targets of SDR7-6.

## Data Availability

The RNA-Seq datasets of the current study are available in the PRJNA1373473 to SRA (Sequence Read Archive) repository http://www.ncbi.nlm.nih.gov/bioproject/PRJNA1373473 (accessed on 4 December 2025).

## Supplementary Materials

The following supporting information can be downloaded at: https://www.mdpi.com/article/10.3390/plants15030490/s1, Figure S1. Relative spectral distributions of light sources used in this study. (A) White light in the growth chamber. (B–D) Monochromatic red, blue, and far red light in growth incubators. All spectra were measured using a spectroradiometer and normalized to their respective maximum intensities to facilitate comparison of light quality independent of absolute photon flux density; Figure S2. Expression patterns of DEGs associated with photosynthesis and chloroplasts; Figure S3. Expression patterns of DEGs associated with GO terms related to red-, blue-, and far-red light responses; Table S1. Primers used in this study.
