# Supplementary material for: Red Light Triggers Lesion Formation in the sdr7-6 Mutant of Rice
Source: Plants (Basel). 2026 Feb 5;15(3):490. doi: 10.3390/plants15030490 (PMC12899952; doi:10.3390/plants15030490)
Supplement: Supplementary file 1 [file plants-15-00490-s001.zip › Supplementary figures.pdf]

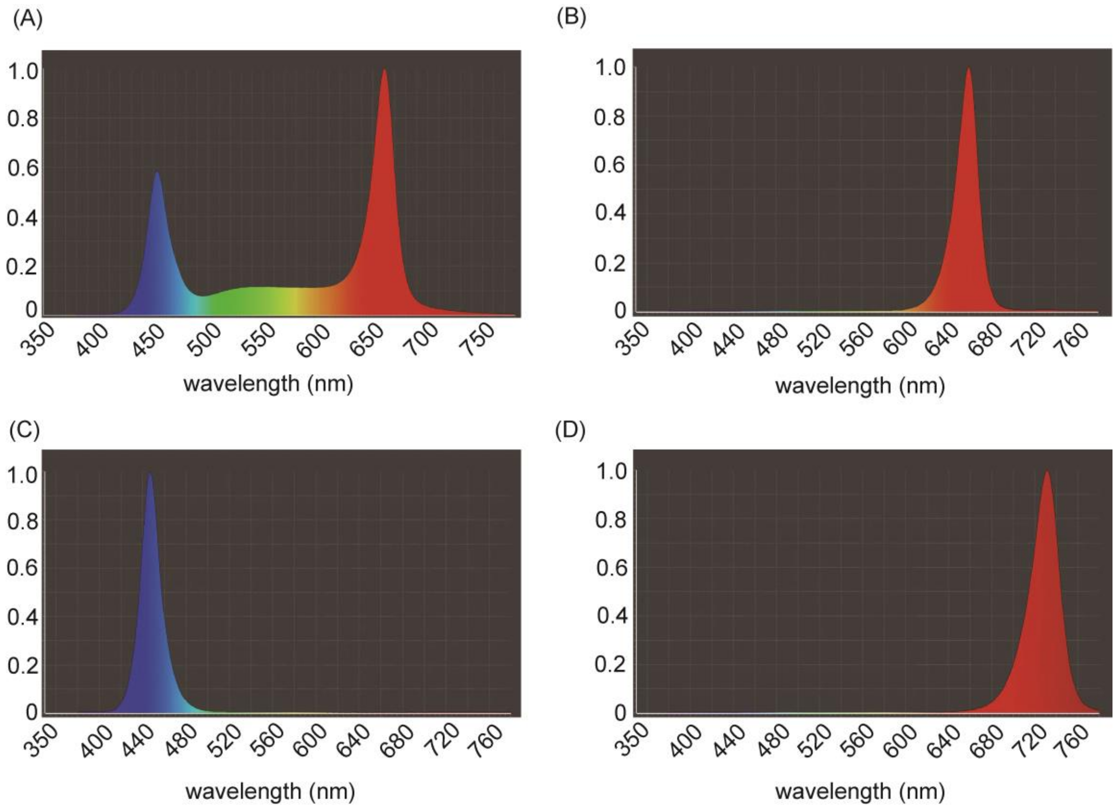

Figure S1. Relative spectral distributions of light sources used in this study. (A) White light in the growth chamber. (B-D) Monochromatic red, blue, and far-red light in growth incubators. All spectra were measured using a spectroradiometer and normalized to their respective maximum intensities to facilitate comparison of light quality independent of absolute photon flux density.

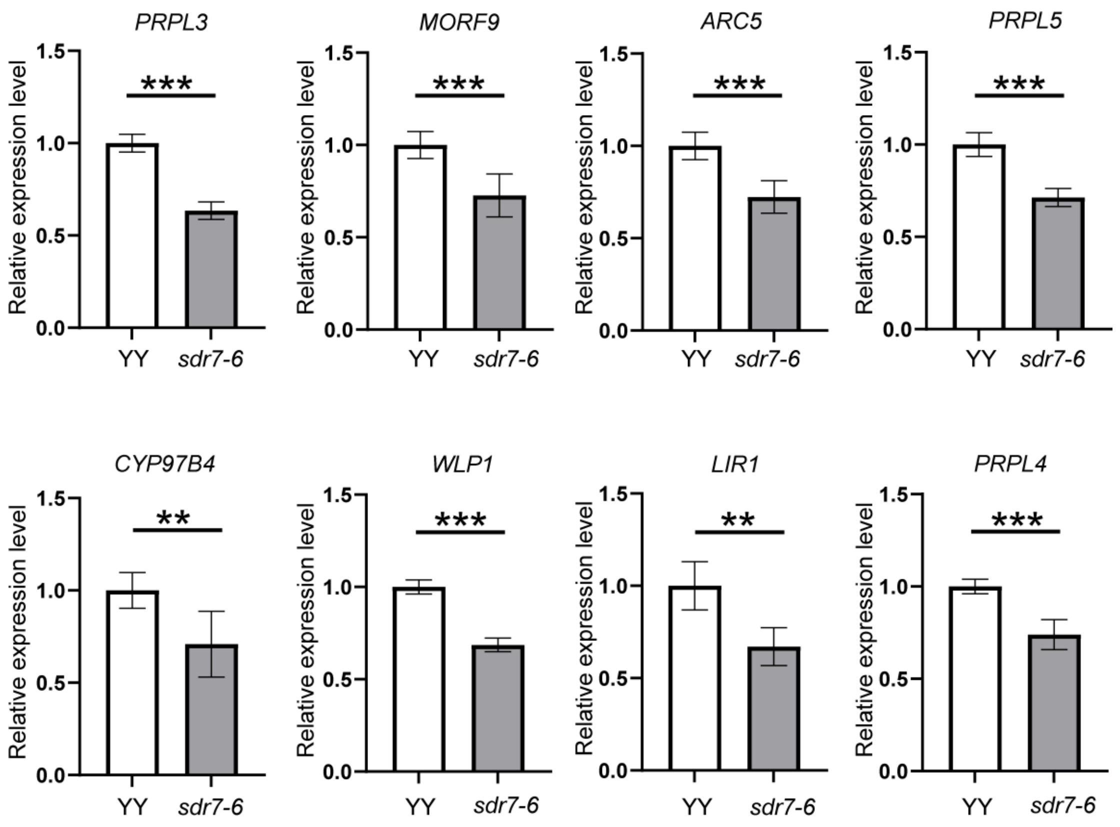

Figure S2. Expression patterns of DEGs associated with photosynthesis and chloroplasts. Data are presented as means ± SD. Statistical significance was determined by Student's *t*-test (**P < 0.01; ***P < 0.001)

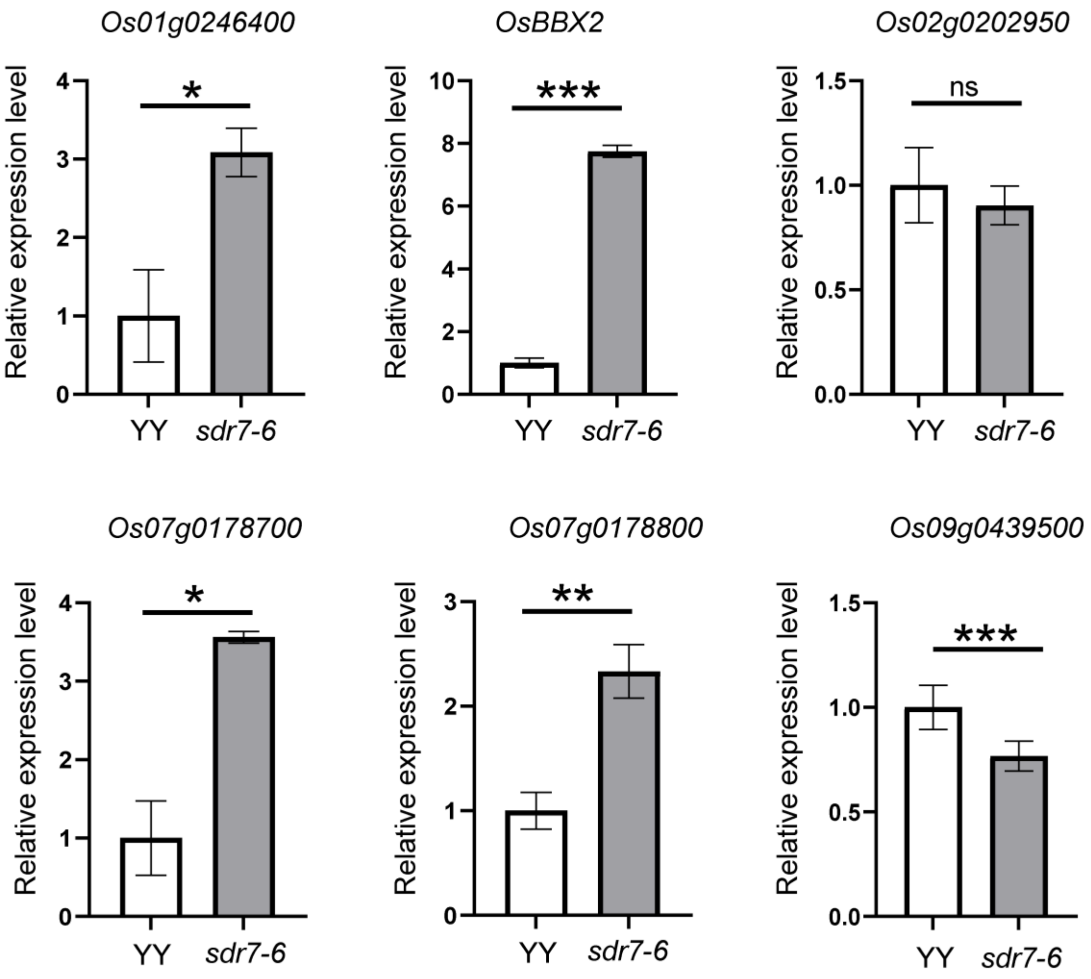

Figure S3. Expression patterns of DEGs associated with GO terms related to red, blue, and far-red light responses. Data are presented as means ± SD. Statistical significance was determined by Student’s *t*-test (*P < 0.05; **P < 0.01; ***P < 0.001; ns, not significant).
