# Supplementary material for: Red Light Triggers Lesion Formation in the sdr7-6 Mutant of Rice
Source: Plants (Basel). 2026 Feb 5;15(3):490. doi: 10.3390/plants15030490 (PMC12899952; doi:10.3390/plants15030490)
Supplement: Supplementary file 1 [file plants-15-00490-s001.zip › TABLE S1 Primers used in this study.pdf]

TABLE S1 Primers used in this study

Primer name	Forward primer	Reverse primer	Reference
phyA-3 CR	TGGCGATGTCCGACATATGGTTTTAGAGCTAGAAAT	CATATGTCGGACATCGCCATGCCACGGATCATCTGC	
phyB-3 CR	TTCGAGCAGTCGGGCGCGTCGTTTTAGAGCTAGAAAT	GACGCGCCCGACTGCTCGAACAACACAAGCGGCAGC	
phyC-3 CR	CAAGCCATGCCCCAGAGCAGTTTTAGAGCTAGAAAT	TGCTCTGGGGCATGGCTTGCGGCAGCCAAGCCAGCA	
2 CR	AATAATGGTCTCTGGCGGAGAGGTTCCCACTGGGCAGTTTTAGAGCTAGAAATAGC	ATTATTGGTCTCTAAACCTGAGTATAGTATCAGGGACGCTTCTTGGTGCC	
PRPL3	CTCGACTTCGCCGACATGTTCAAG	TTGTGCCTCTTGATGCCACCTTGG	
CYP97B4	CAACGCAAGTTCCACAGTGACCTC	AACCTCAGCAAGCTGGCATCCTTC	
PRPL5	GCGGCAGGGTAATGTTCAACTTC	AGTTGCCATGGCCATCAAAGCTG	
OsMORF9	AGTCAGCCTCTTCTTCATGAGGTC	TCTGTCAGAGCTTTCCCGTATGC	
WLP1	GTAGGCTTGCATCCACCATTGC	AGCAACCTTCTCTGCATTAACCAC	
PRPL4	GAGGAGTTGCAAGAAGGAGAAGGG	TCAGTCTCTGCAGCCTCATCTTG	
ARC5	GCACCGCCTGATAGATTTGGAG	ATGGTATTGTGCGCCACCGTAG	
Os01g0246400	GATGGTGGGGTTCGTGTCG	GGTGAGGAACTCGGTGAAGG	
Os02g0176000	CTGACTCCGCGTTTCTTTGC	CTCATCCACCACCTCAACCC	
Os02g0202950	GAGCACAAGCGCCTCAAAAG	TTCAGAAGAACTGGCCCTCT	
Os07g0178700	GATGGTGGGGTTCGTGTCG	CGGTGAGGAACTCGGTGAAG	
Os07g0178800	GATGGTGGGGTTCGTGTCG	GGTGAGGAACTCGGTGAAGG	
Os09g0439500	CGGGAGACTTCGGTTTCGAT	GAACCCCCATTTCTCCAGCA	
Actin	AGTGTCTGGATTGGAGGAT	TCTTGGCTTAGCATTCTTG	
